# Potential causes and significance of elevated blood ketone levels in patients with heart failure with preserved ejection fraction

**DOI:** 10.3389/fnut.2025.1678905

**Published:** 2026-01-12

**Authors:** Weijie Lu, Ai Liu, Mengyang Liu, Yujie Hu, Kang Yang, Yaoting Deng, Qianrong Li, Bowen Wang, Yanling Li, Bing Jiang, Gang Wang, Xuehan Wang, HuGang Jiang, Ping Xie

**Affiliations:** 1 Department of Integrative Chinese and Western Medicine, Gansu University of Traditional Chinese Medicine, Lanzhou, China; 2 Department of Clinical Medicine, Lanzhou University, Lanzhou, China; 3 Department of Cardiology, Gansu Provincial People’s Hospital, Lanzhou, China

**Keywords:** assessment value, blood ketones, clinical research, diagnostic value, heart failure with preserved ejection fraction

## Abstract

**Background:**

The diagnosis of heart failure with preserved ejection fraction (HFpEF) remains challenging. Given the critical role of metabolic disturbance and energy expenditure in HFpEF pathophysiology, we investigated the clinical significance and diagnostic value of blood ketone bodies in these patients.

**Methods:**

This case–control study enrolled 160 participants, comprising 80 HFpEF patients and 80 matched healthy controls. Baseline characteristics, levels of blood ketones (acetoacetate, β-hydroxybutyrate, acetone), and NT-proBNP were compared. Multivariate linear regression and correlation analyses were employed to assess the associations between ketone levels, clinical parameters, and NT-proBNP. The diagnostic performance was evaluated using receiver operating characteristic (ROC) curve analysis.

**Results:**

Compared to controls, HFpEF patients showed significant differences in age, heart rate, BMI, and blood pressure. Multivariate regression revealed a significant linear association between BMI, systolic blood pressure, and acetoacetate levels in the HFpEF group. A weak inverse correlation was found between acetoacetate and NT-proBNP levels. However, no correlation was observed between ketone levels and NYHA functional class. ROC analysis demonstrated that the combination of acetoacetate and NT-proBNP yielded the highest diagnostic efficacy (AUC = 0.9117), superior to NT-proBNP alone (AUC = 0.8328) or any ketone body alone.

**Conclusion:**

Unlike nutritional ketosis, elevated blood ketone levels in patients with HFpEF likely reflect impaired metabolic efficiency rather than a marker of cardiac function. Nevertheless, this phenomenon has diagnostic significance: combining acetoacetate with NT-proBNP can markedly improve diagnostic performance.

## 1 Introduction

Heart failure with preserved ejection fraction (HFpEF) refers to a type of heart failure characterized by a left ventricular ejection fraction of ≥50%, accounting for approximately 50% of all heart failure cases. With the increasing prevalence of predisposing conditions such as aging, obesity, diabetes, hypertension, and chronic kidney disease, HFpEF has become increasingly common among patients with heart failure. Although there are no definitive epidemiological data available domestically, it is estimated that about 3 million people in the United States and as many as 32 million worldwide suffer from HFpEF (1, 2). On average, patients are hospitalized approximately 1.4 times per year, with an annual mortality rate of around 15%, posing a serious threat to human health and life (3). At present, HFpEF is recognized as a distinct clinical entity from heart failure with reduced ejection fraction (HFrEF). While HFrEF is primarily characterized by impaired myocardial contraction (systolic dysfunction), HFpEF is dominated by abnormal myocardial relaxation and increased stiffness (diastolic dysfunction). This distinction is critical because the proven pharmacological therapies for HFrEF have largely failed to improve outcomes in HFpEF, underscoring the necessity for a differentiated diagnostic approach to develop targeted management strategies for each phenotype (4). Despite improvements in our understanding of HFpEF pathophysiology and the developments of more sophisticated imaging modalities, diagnosis remains difficult, particularly when there is chronic progression, and symptoms are induced by exertion; however, diagnostic assessment is primarily performed at rest, inevitably leading to a bias in diagnosis (5, 6). Additionally, owing to the close association between metabolic syndrome (MetS) and obesity and the fact that N-terminal B-type natriuretic peptide (NT-proBNP), a key diagnostic marker for heart failure, has decreased diagnostic sensitivity in patients with obesity (7, 8), the diagnostic value of NT-proBNP for HEpEF is further constrained. Therefore, exploring sensitive and specific tests is vital for the early diagnosis and prognosis of patients with HFpEF.

During enhanced fat mobilization, the body produces blood ketones, including acetoacetic acid, β-hydroxybutyric acid, and acetone. Studies have demonstrated that patients with stable or acutely decompensated HF exhibit increased ketone bodies in urine (9), respiration (10), and plasma (11) compared with healthy controls. Increased ketone body oxidation is associated with higher circulating ketone levels in patients with HF in human and animal HF models (12). Studies have reported that exhaled breath acetone (EBA) exhibits high sensitivity and specificity (85%) in chronic HF diagnosis, with a diagnostic value comparable with that of B-type natriuretic peptide (BNP) and NT-proBNP (13). However, as a novel biomarker, EBA still has several limitations. For instance, its detection methods have not yet been standardized; there is a lack of uniform gas collection procedures (such as standard protocols for cleaning and storing Tedlar sampling bags) and evaluation criteria for results. In addition, detection is easily influenced by other volatile gases or variations in blood volume. Furthermore, results are susceptible to confounding factors, such as comorbidities including diabetes and renal insufficiency, or medication use that may interfere with the specificity of EBA. This study did not include an in-depth analysis of these populations (14). Nevertheless, it reflects the increased concentration of ketone bodies. Gouzi et al. (15) reported that EBA concentration was four times higher in patients with chronic HF than in the general population and up to 10 times higher in patients with significant circulatory stasis. It is noteworthy that a community-based cohort study found that ketone body levels in most individuals were comparable to those in healthy adults (16). However, metabolomics studies have shown that circulating ketone levels are elevated in both HFpEF and HFrEF patients (17). Zordoky et al. (18) found that blood ketone levels in HFpEF patients were significantly higher than those in HFrEF patients. Nevertheless, a study that measured arteriovenous differences in blood ketone concentrations between patients with heart failure and healthy individuals found that blood ketone levels were higher in patients with heart failure than in healthy controls, with a marked increase in cardiac ketone consumption in HFrEF; in contrast, HFpEF appears to exhibit a different pattern of ketone metabolism, which requires dedicated studies to elucidate (18). In the present study, by comparing blood ketone levels between HFpEF patients and healthy individuals, we sought to clarify their diagnostic and evaluative value in HFpEF and, using statistical methods such as regression analysis, to explore the reasons for elevated blood ketone levels. The body’s energy metabolism is closely correlated with changes in blood ketone levels, which are chemicals that participate in this process. Theoretically, comorbidities in HFpEF, such as hypertension, hyperlipidemia, and diabetes, may activate the renin-angiotensin-aldosterone system (RAAS) or disrupt the body’s energy metabolism, which is followed by an increase in fatty acid catabolism and a corresponding increase in blood ketones. However, *in vivo*, the myocardium exhibits the highest level of ketolytic enzyme activity (20), which can increase ketone body utilization to improve the status of myocardial energy metabolism (21). Furthermore, blood ketones can exert cardioprotective effects by modifying mitochondrial function (22), decreasing oxidative stress, and altering post-translational modifications (23). As a result, when cardiomyocytes in patients with HF have poor energy metabolism, secondary activation of RAAS, substrate usage modification, and cardioprotective mechanisms can be used to enhance the status of energy metabolism. The assessment and diagnosis of HFpEF, whether complicated by comorbidities associated with HFpEF or resulting from impaired myocardial energy metabolism, is undertaken from the perspective of different ventricular wall pressure changes, which cover areas that are not covered by NT-proBNP and theoretically improve the assessment and diagnostic efficacy of HFpEF.

In this study, we measured and compared blood ketone levels between patients with HFpEF and healthy controls, and compared baseline data between the two groups to explore potential reasons for elevated blood ketones in HFpEF patients and to determine whether blood ketones could serve as a novel indicator to improve the diagnostic accuracy of HFpEF and to assess cardiac function in HFpEF.

## 2 Materials and methods

### 2.1 Study subjects

This study was approved by the Ethics Committee of Gansu University of Traditional Chinese Medicine (GUSTCM). Eighty patients with HFpEF who visited the Department of Cardiology at GUSTCM and the First Hospital of Lanzhou University from May 2022 to February 2023 were enrolled in the HF group. Furthermore, 80 healthy individuals who visited for medical checkups were enrolled in the control group. Each subject signed the informed consent form. Preliminary results indicate that the sensitivity and specificity of breath acetone for chronic heart failure are 72.6 and 84.6%, respectively. Assuming a two-sided test with both indices allowing a 10% margin of error and a significance level (*α*) of 0.05, the minimum required sample size per group was calculated to be 77 cases; therefore, 80 cases meet the statistical requirements. The calculation formula is as follows:


$$n={\left(\frac{Z_{1-\alpha /2}\times \sqrt{p\times (1-p)}}{\delta }\right)}^{2}$$

### 2.2 Methodology

Information such as age, sex, body mass index (BMI), heart rate, systolic blood pressure (SBP), diastolic blood pressure, New York Heart Association (NYHA) classification, and disease history (hypertension, coronary artery disease, diabetes mellitus, atrial fibrillation, and hyperlipidemia) was collected. Cardiac ultrasonography indices were measured using the GE Vivid7 color Doppler echocardiograph (result obtained from a back-to-back examination conducted by a senior physician in the Echocardiography Laboratory of the Department of Cardiology, First Hospital of Lanzhou University). NT-proBNP measurements were performed on the day of admission. For blood collection, use yellow serum separator tubes containing inert separation gel. After the patient has fasted and rested, collect blood from an antecubital vein; tourniquet application time should be less than 1 min. Immediately after collection, gently invert to mix. Within 30 min, centrifuge the samples at 2–8 °C and 1,500 × g for 10 min. Aliquot the separated serum and store at −80 °C; repeated freeze-thaw cycles are strictly prohibited. After blood collection from all patients was completed, samples were thawed at one time, and serum acetone levels were measured using a Nexis GC-2030 gas chromatograph; acetoacetic acid and β-hydroxybutyric acid were measured using human acetoacetic acid and β-hydroxybutyrate enzyme immunoassay kits, respectively.

### 2.3 Diagnosis and inclusion and exclusion criteria

#### 2.3.1 Diagnostic criteria

The diagnostic standards outlined in the 2018 Chinese Guidelines for the Diagnosis and Treatment of Acute and Chronic Heart Failure were followed (24):(1) HF-related symptoms and indicators; (2) left ventricular ejection fraction >50%; and (3) elevated natriuretic peptide accompanied by at least one of the following: (A) left atrial enlargement or left ventricular hypertrophy, and (B) heart’s abnormal diastolic function. Indications of elevated natriuretic peptide included BNP levels higher than 35 ng/L and/or NT-proBNP levels higher than 125 ng/L. Left ventricular mass index ≥115 g/m^2^ (men) or 95 g/m^2^ (women) was an indicator of cardiac structural abnormalities in HEpEF. The most important warning signs of impaired diastolic function were *E*/*e*′ ≥ 13 and *e*′ mean (septum and free wall) of 9 cm/s. Another two indicators were longitudinal strain or tricuspid regurgitation procedures.

#### 2.3.2 Inclusion criteria

HFpEF patients first diagnosed in the outpatient setting who have not received guideline-directed treatment.

#### 2.3.3 Exclusion criteria

(1) Pregnant women; (2) patients with chronic renal insufficiency; (3) those with chronic liver insufficiency; (4) Continuous use of glucocorticoids for more than 4 weeks; and (5) those with heart valve disease.

### 2.4 Statistical analysis

SPSS 26.0 was used to statistically analyze the data. Measured data were expressed as mean ±standard deviation ( $\bar{x}\pm s$ ). When they followed a normal distribution, the independent samples *t*-test was performed to compare the two samples. Multivariate or univariate regression analyses were performed if statistically significant differences were observed between the baseline data and blood ketone levels of the groups. Count data were expressed as a frequency, and the chi-squared test was performed for comparisons between two samples. If a statistically significant difference was observed between two samples, Spearman’s correlation analysis was performed to elucidate the relationship between two variables in the hierarchical variable. Areas under the curves (AUCs) were calculated to determine the diagnostic sensitivity and specificity of the receiver characteristic curves (ROCs) of the subjects with acetoacetic acid, β-hydroxybutyrate, acetone with NT-proBNP, and blood ketone combined with NT-proBNP for HFpEF diagnosis.

## 3 Results

### 3.1 Statistics of basic information

No statistically significant differences in sex were observed between the HF and control groups (*p* > 0.05). However, age, heart rate, BMI, systolic and diastolic blood pressure, and comorbidities were all statistically different between both groups (*p* < 0.05; Table 1).

**Table 1:** Comparison of the baseline data of the heart failure and control groups.

Variables	HF	Control	*p*-value
Age (mean ± SD, years)^b^	62.213 ± 9.357	56.360 ± 13.229	<0.01
Male [case (%)]^a^	42 (52.5)	40 (50)	0.752
Woman [case (%)]^a^	38 (47.5)	40 (50)	
Heart rate (mean ± SD, bpm)^b^	79.33 ± 7.38	72.88 ± 7.07	<0.01
BMI (mean ± SD, kg/m^2^)^b^	24.48 ± 2.65	22.67 ± 1.96	<0.01
Systolic blood pressure (mean ± SD, mmHg)^b^	132.163 ± 14.009	127.175 ± 10.329	0.011
Diastolic blood pressure (mean ± SD, mmHg)^b^	81.788 ± 9.137	76.563 ± 7.882	<0.01
Hypertension [case (%)]^a^	22 (27.50%)	3 (3.75%)	<0.01
AF [case (%)]^a^	14 (17.50%)	1 (1.25%)	<0.01
Coronary artery disease [case (%)]^a^	18 (22.50%)	2 (2.50%)	<0.01
Diabetes [case (%)]^a^	12 (15.00%)	1 (1.25%)	<0.01
Hyperlipidemia [case (%)]^a^	14 (17.50%)	1 (1.25%)	<0.01

a Performed chi-square test, expressed as frequency (constitutive ratio).

b Performed independent samples *t*-test, expressed as ( $\bar{x}\pm s$ ); heart failure group vs. control group ^*^p < 0.05 and ^#^*p* < 0.05.

### 3.2 Comparison of ketone body levels in the HF and control groups

**Figure 1:**
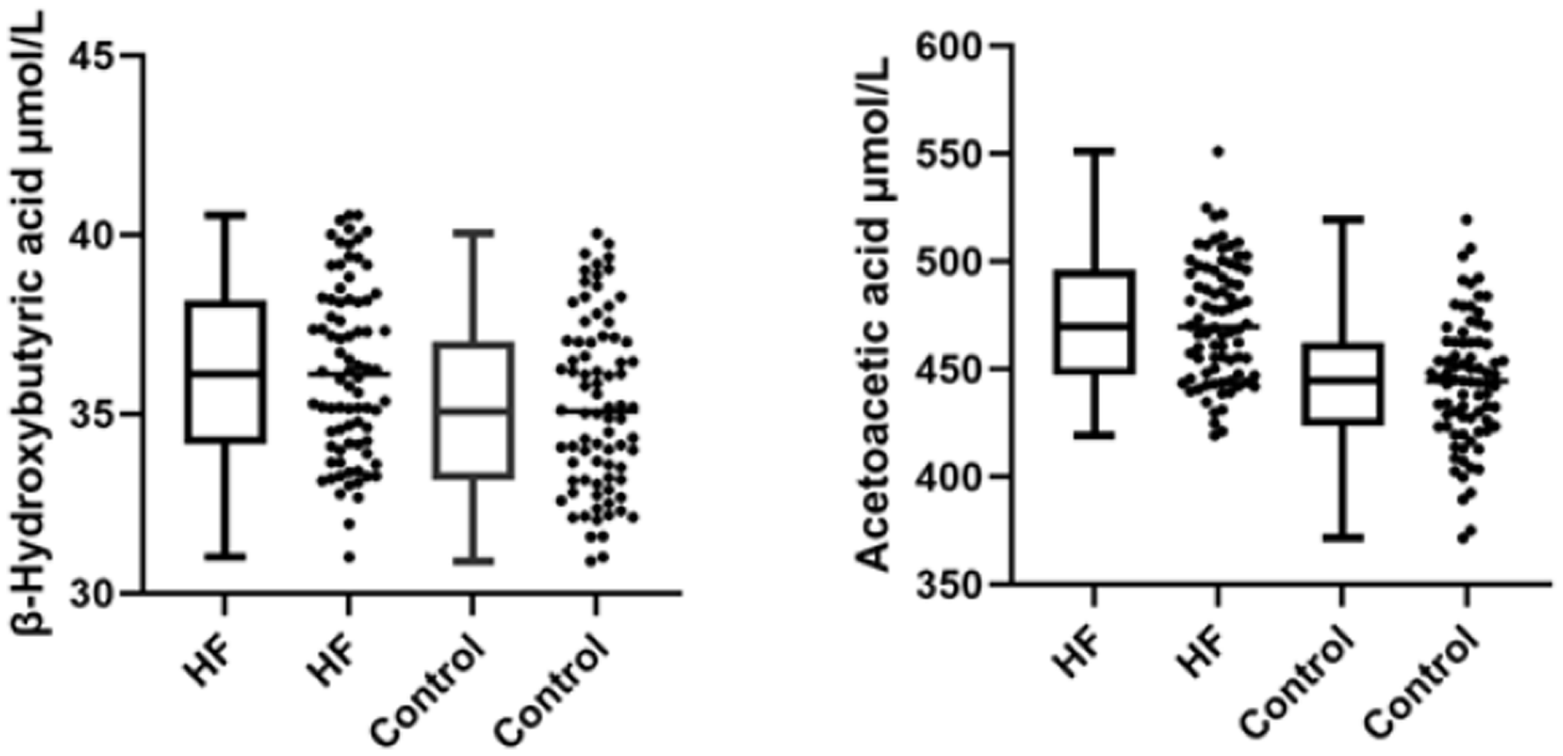
Box and scatter plots of the distribution of β-hydroxybutyric acid and acetoacetic acid between both groups.

In this study, blood ketone body levels were compared between the heart failure (HF) group and the control group. The results showed significant differences in acetoacetic acid and β-hydroxybutyric acid between the two groups, while acetone was not detected. An independent-samples t-test indicated that the β-hydroxybutyric acid level in the HF group (M = 36.20, SD = 2.41 μmol/L) was significantly higher than that in the control group (M = 35.23, SD = 2.40 μmol/L), *t* = 2.54, *p* < 0.05. Similarly, the acetoacetic acid level in the HF group (M = 472.85, SD = 30.49 μmol/L) was significantly higher than that in the control group (M = 443.71, SD = 29.41 μmol/L), *t* = 6.26, *p* < 0.01 (Figure 1).

### 3.3 Multivariate linear regression analysis of age, BMI, heart rate, SBP, and blood ketone levels in the HF group

**Figure 2:**
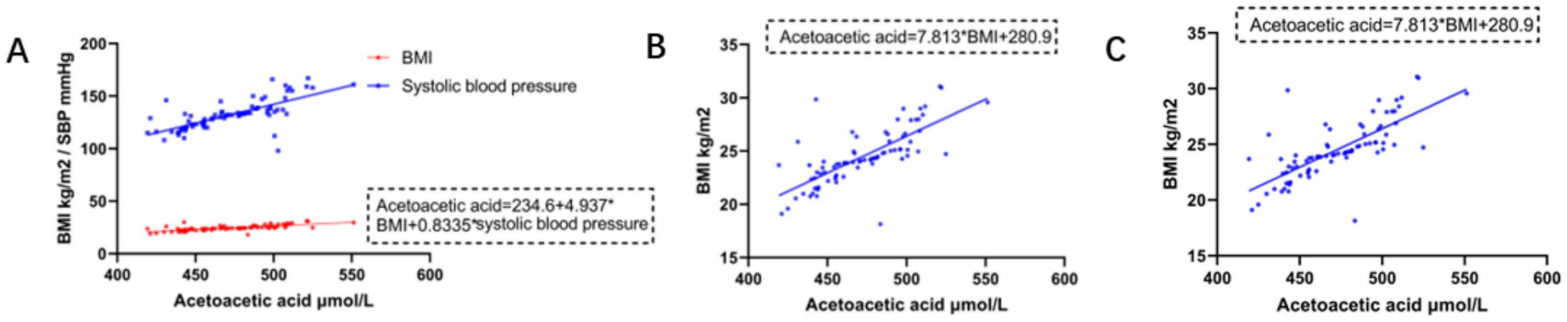
Effects of BIM and SBP on acetoacetate concentration. **(A)** Multivariate regression model of body mass index, SBP, and acetoacetic acid. **(B)** Univariate linear regression model of BMI and acetoacetic acid. **(C)** Univariate linear regression model of SBP and acetoacetic acid.

Multivariate linear regression analysis of age, BMI, heart rate, and SBP with β-hydroxybutyrate did not yield meaningful results. In the HF group, increased acetoacetic acid linearly correlated with BMI, heart rate, and SBP (Figure 2A). Owing to the covariance between heart rate and SBP, the final model included BMI and SBP as independent variables to predict acetoacetic acid levels. The multivariate linear regression model was statistically significant (*F* = 74.936, *p* < 0.01). It explained a substantial proportion of the variance in acetoacetic acid levels, as indicated by an adjusted *R*^2^ of 0.652. Analysis of the coefficients revealed that acetoacetic acid levels increased by 4.937 units (*B* = 4.937, *β* = 0.464) for every 1-unit increase in BMI and by 0.884 units (*B* = 0.884, *β* = 0.440) for every 1-unit increase in SBP, with both predictors being significant contributors (*p* < 0.01). The model met the key assumptions of regression: the variance inflation factor (VIF) for both predictors was 1.608, indicating no multicollinearity, and the Durbin–Watson statistic of 2.033 confirmed the independence of observations.

### 3.4 Univariate linear regression analysis of BMI and acetoacetic acid in the HF group

In the HF group, a substantial linear association was observed between BMI and acetoacetic acid, i.e., acetoacetic acid levels increased by 7.813 for every 1 unit increase in BMI; BMI was responsible for 54% of the total variance in acetoacetic acid (the dependent variable) in the HF group (Figure 2B and Table 2). The residuals conformed to normal distribution, and the results were statistically significant.

**Table 2:** Data for univariate regression analysis of BMI and acetoacetic acid.

Variables	*F* value	Significance	Unstandardized coefficient	Standardized coefficient	*R* ^2^
BMI	91.625	<0.01	7.813	0.816	0.534

### 3.5 Univariate linear regression of SBP and acetoacetic acid in the HF group

In the HF group, a statistically significant linear association was observed between SBP and acetoacetic acid, i.e., acetoacetic acid levels increased by 1.457 units for every 1 unit increase in SBP. SBP contributed to 52.6% of the total variance in acetoacetic acid (the dependent variable) in the HF group (Figure 2C and Table 3). The residuals generally conformed to normal distribution, and the results were statistically significant.

**Table 3:** Data for univariate regression analysis of SBP and acetoacetic acid.

Variables	*F* value	Significance	Unstandardized coefficient	Standardized coefficient	*R* ^2^
SBP	86.723	<0.01	1.457	0.726	0.526

### 3.6 Correlation analysis of blood ketones and NT-proBNP

**Figure 3:**
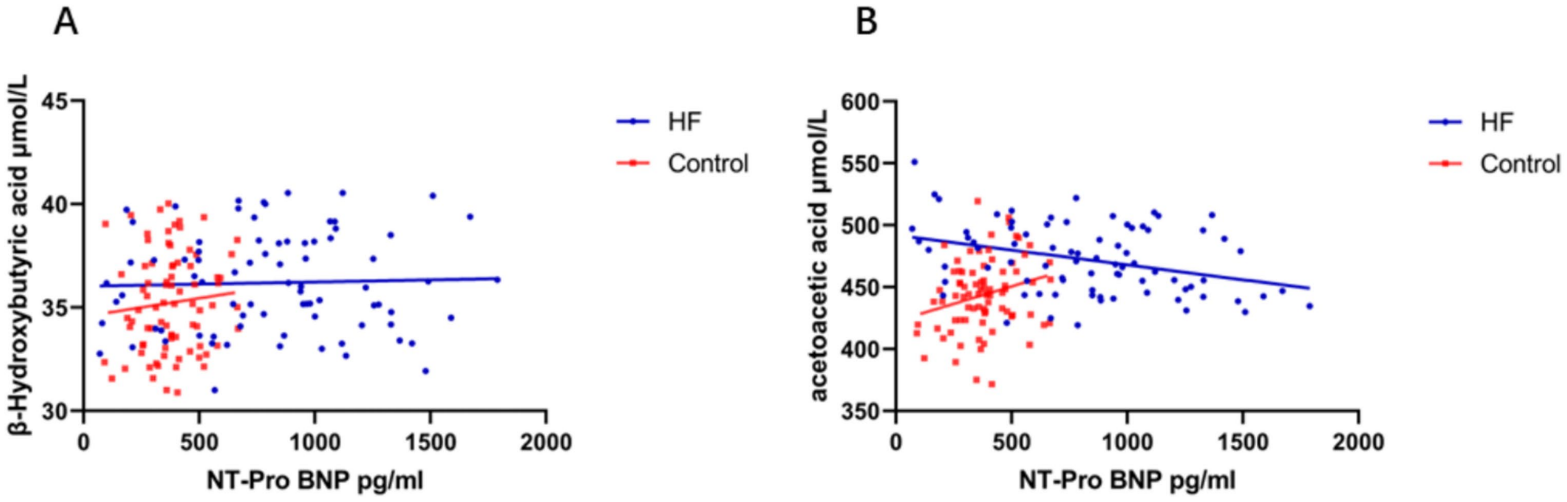
Scatterplot of the correlation between β-hydroxybutyrate/acetoacetic acid and NT-proBNP levels between the two groups. **(A)** Correlation analysis between β-hydroxybutyrate and NT-proBNP levels. **(B)** Correlation analysis between acetoacetate and NT-proBNP levels.

Pearson correlation analyses were conducted to examine the relationships between ketone bodies and NT-proBNP. In the heart failure (HF) group, there was a significant negative correlation between acetoacetic acid and NT-proBNP levels, *r*(78) = −0.348, *p* = 0.002. In the control group, this relationship was a significant positive correlation, *r*(78) = 0.249, *p* = 0.026. However, no significant correlation was found between β-hydroxybutyric acid and NT-proBNP in either the HF group [*r*(78) = 0.034, *p* = 0.762] or the control group [*r*(78) = 0.096, *p* = 0.397]. When all participants were analyzed together without group distinction, neither β-hydroxybutyric acid nor acetoacetic acid showed a significant correlation with NT-proBNP (both *p* > 0.05) (Figure 3).

### 3.7 Correlation between different NYHA classifications and blood ketone (β-hydroxybutyric acid and acetoacetic acid) levels

**Figure 4:**
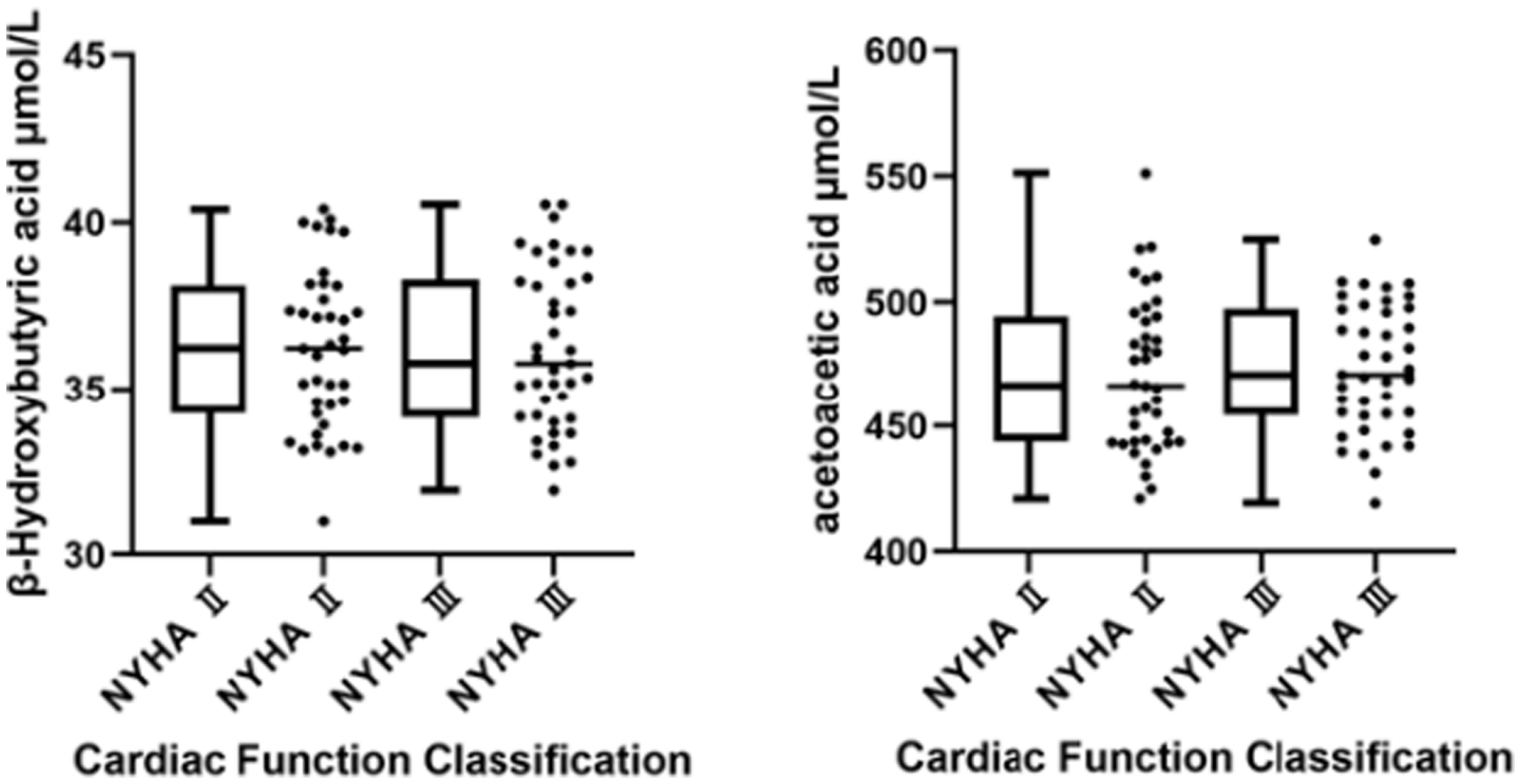
Box and scatter plots of acetoacetic acid and β-hydroxybutyric acid in different cardiac function classifications.

Spearman’s rank correlation analysis was performed to assess the relationship between cardiac function class and blood ketone levels. The results indicated no significant correlation between cardiac function class and the levels of β-hydroxybutyric acid (*r*-values = 0.002, *p* = 0.989) or acetoacetic acid (*r*-values = 0.080, *p* = 0.483) (both *p*-values >0.05, *N* = 80) (Figure 4).

### 3.8 Diagnostic value of blood ketones, NT-proBNP, and their combination in HFpEF

#### 3.8.1 Evaluation of the authenticity of the single diagnostic tests

**Figure 5:**
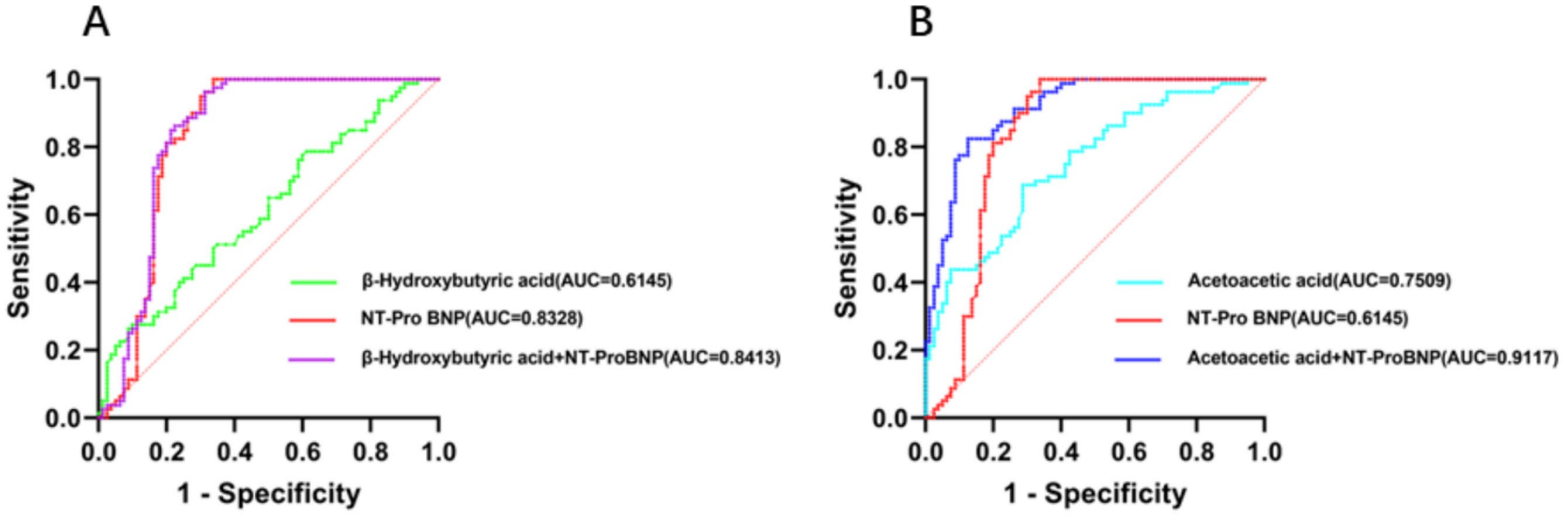
ROC curve. **(A)** ROC curves for the diagnostic values of β-hydroxybutyric acid, NT-proBNP, and their combination. **(B)** ROC curves for the diagnostic values of acetoacetic acid, NT-proBNP, and their combination.

The statistical results of the authenticity evaluation of the diagnostic tests revealed that the sensitivity of β-hydroxybutyric acid, acetoacetic acid, and NT-proBNP was as follows: 0.9125, 0.900, 0.725, 0.6625, 0.4125, 0.400, 0.3875 (note: multiple values were obtained for the sensitivity of β-hydroxybutyric acid), 0.713, and 0.663. The specificity was 0.2625, 0.275, 0.450, 0.5125, 0.7625, 0.775, 0.7875 (note: multiple values were obtained for the specificity of β-hydroxybutyric acid), 0.687, and 1.000. The Youden index was 0.175, 0.400, and 0.663, and AUC was 0.6145, 0.7509, and 0.8328 (Figure 5A).

#### 3.8.2 Evaluation of the veracity of β-hydroxybutyric acid and acetoacetic acid in a combined diagnostic test with NT-proBNP

The statistical results of the authenticity evaluation of the diagnostic test revealed that the sensitivity of β-hydroxybutyric acid combined with NT-proBNP and acetoacetic acid combined with NT-proBNP was 0.963 and 0.875; specificity was 0.687 and 0.825; Youden index was 0.650 and 0.700; and AUC was 0.8413 and 0.9117, respectively (Figure 5B).

## 4 Discussion

### 4.1 Comorbidities in HFpEF may play a role in the high blood ketone levels in patients

Previous studies have reported that blood ketone levels are higher in patients with HF and in patients with HFpEF than with HfrEF (11, 12, 25). However, these investigations only examined total ketone body concentrations and did not address specific changes in β-hydroxybutyric acid, acetoacetic acid, or acetone levels. In the present study, we measured these three substances simultaneously and observed that serum acetoacetic acid and β-hydroxybutyric acid levels were significantly higher in the HF group than in the normal group (*p* < 0.05). However, serum acetone levels were not measured. A community-based heart failure cohort study found no significant difference in blood ketone levels between HFpEF and HFrEF, and levels were comparable to those in healthy adults (16). This differs from our study and from prior studies; the possible reasons include selection bias and the methods used to measure blood ketones. First, patients diagnosed at their first hospital admission are often more severely ill, whereas patients in heart failure follow-up cohorts typically receive standardized treatment. Similar to the heart failure diagnostic biomarker BNP, blood ketone levels may rise during the acute admission period and improve markedly after standardized treatment. Second, this cohort study used NMR spectroscopy to measure three ketone bodies (β-hydroxybutyrate, acetoacetate, and acetone) simultaneously and calculated their total; this calculation differs from ours and is another potential reason for the discrepant results. Therefore, to clarify whether blood ketone levels differ between HFpEF and HFrEF, it is preferable to enroll newly diagnosed, hospitalized heart failure patients who have not yet received standardized treatment, thereby better avoiding the confounding effects of medications. Blood ketones should also be measured using a unified approach to minimize bias introduced by different measurement methods.

Baseline characteristics such as age, BMI, heart rate, SBP, diastolic blood pressure, and comorbidities were different. The abovementioned parameters may increase blood ketone levels. With an adjusted *R*^2^ of 0.661, multivariate linear regression analysis revealed a correlation among BMI, SBP, and acetoacetic acid increase. This revealed that 66.1% of the increase in acetoacetic acid levels was caused by changes in BMI and SBP. Univariate linear regression analysis of BMI, SBP, and acetoacetic acid revealed a significant linear relationship between BMI and acetoacetic acid (*R*^2^ = 0.540) and between SBP and acetoacetic acid (*R*^2^ = 0.526) to further clarify the extent of the effect of BMI and SBP on the increase in acetoacetic acid levels. The abovementioned findings suggest that higher SBP (representing pressure load) and BMI (representing metabolic burden) are both independent predictors of higher acetoacetic acid levels, and their effects are of similar magnitude.

Age, BMI, heart rate, SBP, diastolic blood pressure, and comorbidities did not significantly correlate with β-hydroxybutyric acid; this may be related to the function of β-hydroxybutyric acid dehydrogenase, an important enzyme in the conversion of acetoacetic acid to β-hydroxybutyric acid. This relationship deserves further investigation. Another issue concerning β-hydroxybutyrate is that the enzyme-linked immunoassay kits used to measure its concentration cannot distinguish between the R- and S-forms of β-hydroxybutyrate. Since R-β-hydroxybutyrate is the predominant active form in human metabolism, while pathological conditions can also produce the S-form, future studies should place greater emphasis on the relationship between β-hydroxybutyrate subtypes and heart failure. Patients with HFpEF have higher exhaled acetone levels, which exhibit a high diagnostic value for the condition (26). However, serum acetone levels were not assessed in the present study because gas chromatography may not have been able to identify the very low serum acetone level. According to previous determinations of human serum acetone levels using high-performance liquid chromatography (HPLC), the average serum acetone concentration in patients with diabetes is 0.1318 mmol/L. However, other studies have indicated that most diabetic patients maintain normal ketone levels when in a stable condition, while the average concentration in healthy individuals is 0.0856 mmol/L (27–29). In general, 1 mmol/L translates to 1 μg/mL/M (M stands for mass of substance). This conversion formula can be used to observe how much lower the acetone levels recorded by gas chromatography are than those measured using HPLC (in μg/mL). Acetone cannot be quantified using gas chromatography primarily because the instrument is not suitable for analyzing trace components.

Blood ketone levels are affected by meal status, for example, famine, which results in higher ketone synthesis because of fat mobilization. Furthermore, blood ketone levels are affected by disturbances in glucose metabolism, including decreased glucose catabolism, inhibition of the tricarboxylic acid cycle, and a shift in ketone body production by acetyl CoA in the mitochondria, or a lack of oxaloacetate, which prevents acetyl CoA from entering the tricarboxylic acid cycle and progressing toward ketogenesis. What causes increased blood ketones in patients with HF if blood ketones are synthesized in the liver and the heart has no evident relationship with meal status or glucose metabolism control? The glucose ketone index (GKI) is a single numerical indicator that integrates blood glucose and ketone levels to quantify the body’s metabolic state. It is calculated as GKI = [blood glucose (mg/dL)/18]/blood ketone (mmol/L) (30). Theoretically, a persistently high GKI value reflects systemic insulin resistance and metabolic inflexibility in patients with HFpEF. This may indirectly suggest that similar metabolic distress could also exist in the myocardium. Both SGLT2 inhibitors and ketogenic diets can increase blood ketone levels and improve myocardial energy utilization efficiency (31, 32). Therefore, GKI is expected to serve as a potential biomarker for systemic and myocardial metabolic disorders, as well as a dynamic monitoring tool to assess the effectiveness of emerging metabolic therapies (e.g., SGLT2 inhibitors, ketogenic diet). In particular, is HFpEF superior to HFrEF? The authors believe that elevated blood ketones are closely associated with the formation and comorbidity of HFpEF or that comorbidities in HFpEF lead to elevated blood ketones. Recently, HFpEF has been recognized as a syndrome of comorbidities, including hypertension, diabetes mellitus, obesity, coronary artery disease, chronic kidney disease, atrial fibrillation, anemia, sleep apnea syndrome, cardiac valvular disease, aging, and specific types (myocardial amyloidosis) as well as many other disorders. These disorders exert metabolic or stressful loads on the body, resulting in systemic inflammation and further developing into HFpEF (28). Furthermore, the development of HFpEF may be closely associated with coronary microcirculatory dysfunction, and pathological autopsies have discovered that 75% of patients with HFpEF have microcirculatory dysfunction (33). This is because hypertension, diabetes mellitus, hyperlipidemia, and obesity cause various proinflammatory events that ultimately result in eNOS uncoupling and endothelial dysfunction, including changes in coronary distribution density and structural function, further resulting in HfpEF (34–36). MetS is a collection of metabolic illnesses characterized by hypertension, dyslipidemia, weight gain, and insulin resistance (37). MetS is associated with unfavorable cardiac remodeling, including stiffness and left ventricular hypertrophy, which can lead to cardiac dysfunction, mostly characterized by diastolic dysfunction and HFpEF (7, 8). Regardless of whether MetS, coronary microcirculatory dysfunction, or HFpEF-induced systemic inflammation, the aforementioned diseases appear to be concomitant with HFpEF, including hypertension, diabetes mellitus, hyperlipidemia, and obesity. Theoretically, RASS activation in patients with hypertension leads to fat mobilization and elevated blood ketones. Furthermore, patients with obesity and hypertriglyceridemia have elevated serum free fatty acid levels, with glucose metabolism disorders in patients with diabetes. Because of the negative feedback regulation of glucose–lipid metabolism, mitochondrial acetyl CoA is more prone to a ketogenic shift. Therefore, HFpEF comorbidities such as diabetes mellitus, hypertension, and obesity may contribute to high blood ketones, which may also be predisposed to HFpEF.

Nutritional ketosis can also lead to elevated blood ketone levels, but unlike the increase observed in patients with HFpEF, the underlying mechanisms and metabolic contexts differ significantly. Nutritional ketosis is a physiological and adaptive metabolic state. It is actively induced by exogenous interventions such as a very low-carbohydrate ketogenic diet or fasting. The core mechanism involves restricted carbohydrate intake, leading to decreased insulin levels and increased glucagon levels, thereby activating hepatic fat mobilization and ketone body production to replace glucose as an energy source for organs such as the brain (38). In this state, the body’s glucose and lipid metabolic regulatory systems are intact and functionally normal. In contrast, the elevation of ketones in HFpEF patients is more likely to represent a pathological and stress-related metabolic alteration. Its trigger is not insufficient carbohydrate intake, but rather complications closely associated with HFpEF as a systemic metabolic inflammatory syndrome. Our multivariate linear regression analysis indicates that BMI and SBP are independent factors influencing the rise of acetoacetate levels. Furthermore, the relationship between ketone levels and energy metabolism efficiency differs markedly between the two conditions. In nutritional ketosis, elevated ketone levels signify a highly efficient metabolic shift toward using fat and ketone bodies as the primary fuel sources. Ketones serve as efficient energy substrates and are typically accompanied by improved mitochondrial function and energy utilization efficiency, providing protective effects on the heart and brain. In contrast, studies including the present one have shown that HFpEF patients exhibit myocardial energy metabolism disorders. Despite elevated blood ketone levels, cardiomyocytes may be unable to effectively utilize these ketones, reflecting a state of “fuel starvation” within the myocardium. The elevation of ketones in this context may represent a compensatory mechanism, but it actually indicates impaired myocardial energy metabolism efficiency—opposite to the enhanced metabolic flexibility seen in nutritional ketosis. Although the mechanisms underlying ketone elevation differ, studies have found that ketogenic diets and exogenous ketone salts can improve the clinical phenotype in HFpEF rat models (39). Therefore, whether a ketogenic diet could serve as a potential therapeutic approach for HFpEF warrants further clinical investigation.

### 4.2 Ketone body levels cannot be used to assess how superior the cardiac performance of HEpEF

In patients with non-ischemic HF, elevated exhaled acetone levels are associated with the severity of the condition and volume overload rather than with the contractile function of the heart (40). In the present study, Spearman’s rank correlation analysis of acetoacetic acid, β-hydroxybutyric acid, and various heart functions revealed no linear relationship between blood ketone and cardiac function levels. Furthermore, Pearson’s linear association analysis revealed the negative correlation between NT-proBNP and acetoacetic acid. The American College of Cardiology, American Heart Association, American Heart Failure Society, and others have frequently recommended NT-proBNP and BNP as evaluation indices for HF diagnosis and prognosis (4). However, no correlation has been observed between β-hydroxybutyric acid and NT-proBNP, or a negative correlation has been observed between acetoacetic acid and NT-proBNP, suggesting that blood ketone levels cannot be used as a prognostic indicator for HF.

At present, impaired active diastolic capacity and passive diastolic function are the primary characteristics of HFpEF pathophysiology. The latter comprises the limitation of passive diastolic function caused by the thickness of the ventricular wall, myocardial fibrosis, and extracellular matrix deposition, whereas the former depends on myofilament dissociation, which is governed by cytosolic calcium dynamics (41). In other words, variables that can result in extracellular matrix deposition which would restrict diastole or factors that can prevent myofilament dissociation in the mitochondria which would lead myocardial hypertrophy, can accelerate HEpEF evolution. The abovementioned disorders are affected by two primary pathophysiological mechanisms. One mechanism is inflammation, which inhibits NO–sGC–cGMP–PKG, lowers endothelial NO secretion, and decreases the capacity of PKG to govern cellular diastole by controlling agonist protein phosphorylation (42). By decreasing inducible NOS, inflammation can also prevent unfolded proteins from responding, leading to a buildup of unstable proteins in the intercellular matrix (43). The transcription of *α*-smooth muscle actin in intrinsic cardiac fibroblasts is induced by reparative (M2) macrophages during the inflammatory regression phase, increasing myocardial fibrosis, cardiac stiffness, and impaired myocardial diastolic function (44). The second mechanism is ROS-induced oxidative stress, which alters Ca^2+^-adenosine triphosphatase activity, decreases the calcium sensitivity of myofilaments, and induces cardiac fibroblast proliferation and matrix metalloproteinases, ultimately resulting in extracellular remodeling and promoting fibrosis (45). HFpEF development and progression are affected by several factors, including hypertension (46), obesity (47), diabetes mellitus (48), and hyperlipidemia (49), which are caused by stress, metabolic load, lipotoxicity, or the formation of late-stage glycosylation end-products that cause oxidative stress or inflammation. HFpEF progression in terms of this process depends on two factors: the persistence of stimuli that cause inflammation or oxidative stress and the length of the persistence of the abovementioned stimuli, i.e., myocardial hypertrophy, myocardial fibrosis, and extracellular matrix deposition are a gradual accumulation of the abovementioned factors and the gradually increasing severity of HF. Blood ketones, which can be used by the myocardium, brain, and other organs and converted by gluconeogenesis, can also be metabolized by the lungs and kidneys and are elevated in association with hypertension, diabetes mellitus, hyperlipidemia, and other metabolic diseases. Therefore, ketone body levels are maintained at a relatively stable level if there are no short-term drastic changes in glucose and lipid metabolism. Considering this, it makes sense why there is no connection between blood ketone levels and heart function prognosis.

Unexpectedly, NT-proBNP and acetoacetic acid levels are negatively correlated. The demographic from which the cases were gathered may have some plausible explanations. Obesity is associated with decreased BNP and NT-proBNP levels, decreasing their diagnostic sensitivity (50, 51). This is because individuals with obesity have higher amounts of enkephalinase (52); however, enkephalinase does affect NT-proBNP, which should logically also not be low. Therefore, this explanation may not concord well with NT-proBNP. When the cases were gathered, BMI was not taken into account; however, BMI was typically higher in the HF group. As a result, patients with obesity have higher lipids, which are naturally higher-than-normal blood ketone levels, and lower NT-proBNP levels. The target group typically presents with lower NT-proBNP levels when HFpEF is misdiagnosed because the diagnosis is not very accurate. Furthermore, a metabolic disorder with high blood ketones and low NT-proBNP contributes to this unfavorable connection.

### 4.3 Acetoacetic acid combined with NT-proBNP can improve the diagnostic efficacy of HEpEF

The 2022 AHA/ACC/HFSA Guidelines for the Management of Heart Failure state that elevated BNP and NT-proBNP levels help diagnose HFpEF; however, normal levels do not exclude the diagnosis (40). Although invasive kinetics is the gold standard for HFpEF diagnosis, there is no particular treatment strategy for HFpEF, and the risk/benefit balance of invasive investigations is controversial (53). The validation of noninvasive natriuretic peptide and echocardiographic metrics revealed that they only lowly correlate with invasive hemodynamic parameters (54). There is no clear cutoff value for various parameters because most of them are continuously distributed in the population and may vary depending on factors such as age, sex, body surface area, BMI, heart rate, and presence or absence of cardiac and noncardiac symptoms. Furthermore, different diagnostic parameters reflect the different pathomechanisms and different stages of HFpEF (6). Another problem is that left atrial pressure, diastolic echocardiographic index function, and serum natriuretic peptide levels fluctuate over time. Repeated measurements of serum natriuretic peptide revealed a concentration variability of up to 100% (55). Furthermore, echocardiographic measurements are hampered by intra- and inter-observer variabilities. Measurements performed at rest may produce false-negative results in patients with chronic HFpEF because symptoms are observed during physical activity (56). Because a single measurement of a parameter cannot provide a conclusive answer in HFpEF diagnosis, integrated diagnosis is the preferred method. In general, the Youden index should be ≥0.7; otherwise, the value of the clinical operation is poor; furthermore, 0.8 < AUC ≤ 0.9 indicates a high diagnostic value, whereas AUC >0.9 indicates a higher diagnostic value. In the present study, as a diagnostic index for HFpEF, NT-proBNP had an AUC of 0.8328 and a Youden index of 0.663; this suggests it exhibits a strong diagnostic value but limited practical applicability. However, with an AUC of 0.9117 and Youden index of 0.7000, the diagnostic value of acetoacetic acid combined with NT-proBNP as a diagnostic indicator of HFpEF was significantly improved. We hypothesize that individuals with obesity cannot be overlooked and that low NT-proBNP performance in these patients restricts the diagnostic utility of HFpEF. Acetoacetic acid combined with NT-proBNP increases diagnostic efficacy and offers a solution. Elevated acetoacetic acid levels, associated with higher BMI in the HF group, serve as a diagnostic index for HFpEF. This condition includes some overweight or obese patients diagnosed based on NT-proBNP; however, the sensitivity of this acetoacetic acid index is low. This may be why acetoacetic acid combined with NT-proBNP can improve diagnostic efficiency.

### 4.4 Strengths and limitation

First, this study collected human blood ketone levels, which are more sensitive than urinary ketone levels. Second, the combined analysis of blood ketones and NT-ProBNP can improve diagnostic accuracy in obese patients who are less responsive to NT-ProBNP, demonstrating good clinical value. However, although this study collected blood ketone data, dietary habits in western China rarely include “nutritional ketosis diets” (such as the ketogenic diet). Therefore, during the subject recruitment process, no particular attention was paid to whether individuals followed a nutritional ketosis diet, which may have introduced some bias into the results. Meanwhile, compared with the sample size of the cohort study, our sample size is relatively small, and subsequent multicenter studies with larger sample sizes can be conducted.

## 5 Conclusion

Unlike nutritional ketosis, elevated blood ketone levels in patients with HFpEF likely reflect impaired metabolic efficiency rather than a marker of cardiac function. Nevertheless, this phenomenon has diagnostic significance: combining acetoacetate with NT-proBNP can markedly improve diagnostic performance.

## Data Availability

The original contributions presented in the study are included in the article/supplementary material, further inquiries can be directed to the corresponding author.
